# Evaluating carbon affinity in catalytic methane decomposition using UBI-QEP energetics and DFT carbon-binding analysis

**DOI:** 10.1039/d6ra05232k

**Published:** 2026-10-20

**Authors:** Yahya Sorkhe

**Affiliations:** a Department of Metallurgical and Chemical Engineering, Istanbul Technical University Istanbul 34469 Turkey Sorkhe20@itu.edu.tr; b Faculty of Science, Koc University Istanbul Turkey

## Abstract

Catalytic methane decomposition produces hydrogen and solid carbon, but carbon growth does not necessarily cause catalyst deactivation. This study examines the usefulness and limitations of carbon affinity as a descriptor using UBI-QEP energetics for Fe, Ni, Pt and Cu, supported by published density functional theory (DFT) and experimental evidence. The methane-to-adsorbed-carbon enthalpy, *D*_C_, correlates strongly with the carbon chemisorption energy (*r* = −0.994) and almost exactly with the combined carbon and hydrogen binding term, *Q*(C) + 4*Q*(H). These relationships reveal its algebraic dependence on the underlying adsorption energies, rather than establishing an independent descriptor. Closing the thermochemical cycle to gaseous H_2_ eliminates the hydrogen-binding contribution and clarifies the physical meaning of the chosen endpoint. An internally consistent DFT dataset covering 14 transition metals provides an external energetic comparison of carbon-binding order. For the four core metals, carbon-binding magnitudes show strong correlation (*r* = 0.929), although the Ni–Pt ordering is reversed. Facet-dependent adsorption, carbon migration barriers and experimental comparisons further demonstrate why elemental carbon affinity alone cannot predict activity or catalyst lifetime. Neither the DFT energetic comparison nor the heterogeneous experimental contrasts constitute validation of an out-of-sample model for catalytic activity or catalyst lifetime. Graphite-referenced equilibrium calculations quantify temperature and pressure effects without assuming quasi-equilibrium among the adsorbed CH_*x*_ intermediates. The analysis retains *D*_C_ as a transparent semi-empirical reaction-energy index while identifying carbon transport, nucleation and catalyst-state evolution as essential to interpreting filament growth, encapsulation and sustained hydrogen production.

## Abstract 2

UBI-QEP energetics connect methane activation with surface carbon formation across Fe, Ni, Pt and Cu. External DFT benchmarks and carbon-transport pathways link carbon affinity to filament growth, encapsulation and catalyst stability.

## Graphical abstract

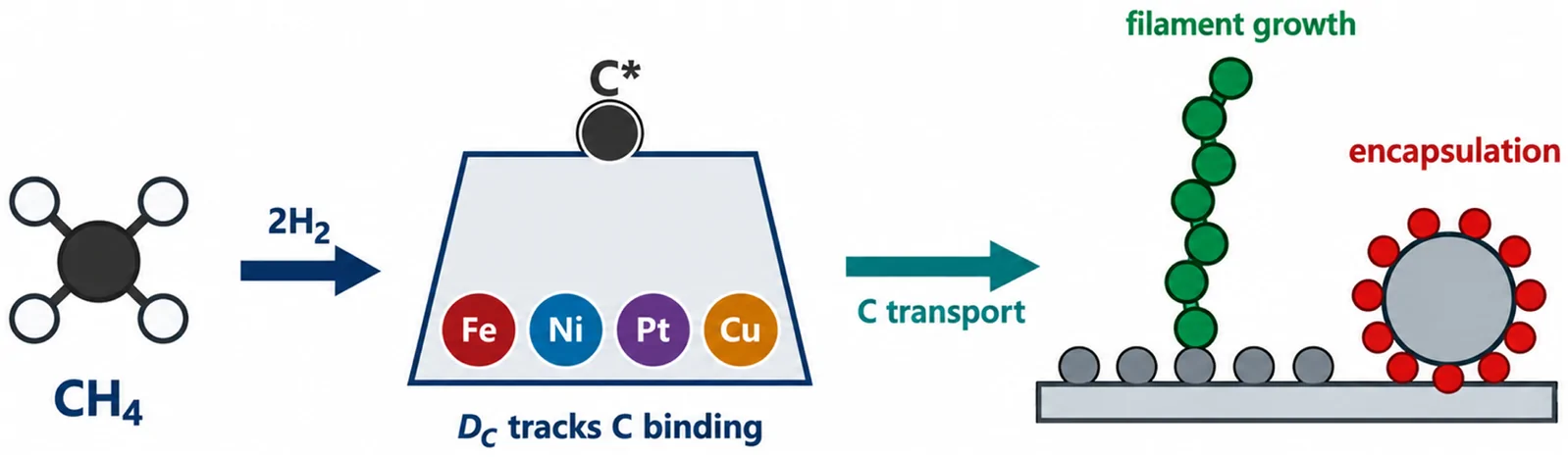

## 1 Introduction

Catalytic methane decomposition (CMD), CH_4_ → C + 2H_2_, produces hydrogen and solid carbon without direct CO_2_ formation in the ideal reaction. Its overall environmental benefit also depends on methane leakage, heat supply and the eventual use of the carbon product.^1–3^ Catalyst selection must address methane activation and continued carbon removal from active ensembles simultaneously. Carbon is the intended coproduct, but an encapsulating layer can suppress reaction even when the total deposited mass is small. Conversely, filament growth may sustain hydrogen production while accumulating substantial carbon. Recent assessments distinguish metal catalysts, carbon catalysts and reactor-dependent performance.^4–6^

The contemporary literature emphasizes catalyst stability and recoverable carbon quality alongside hydrogen production.^7,8^ Carbon-based catalysts constitute a separate class with their own evolving active sites;^9,10^ they are not assigned elemental-metal affinity values in this study.

A carbon-affinity description has a clear physical basis: the metal–carbon interaction influences both the energetics of C–H cleavage and the stability of the resulting carbon-containing species. However, this is not a previously unrecognized connection. CH_*x*_ adsorption scaling with atomic carbon adsorption is established,^11^ as are the broader descriptor and Sabatier frameworks.^12^ Pototschnig *et al.* recently applied a DFT-based model to thirty elements.^13^ A methane to C* quantity must therefore be assessed against carbon adsorption itself, rather than presented as a new independent universal descriptor. General scaling analyses provide the relevant baseline for judging whether a reformulated reaction energy supplies independent information.^14,15^

UBI-QEP provides a compact semi-empirical route to surface reaction energetics.^16^ Its analytic construction is useful for exposing parameter dependence, but consistency within a parameter set does not establish accuracy across metals. A modern DFT dataset can challenge its ordering even when both methods connect fragment stability to carbon binding. Moreover, neither a single adsorption energy nor a total dehydrogenation enthalpy directly specifies the carbon transport and nucleation processes that determine catalyst lifetime. Earlier UBI-QEP work also treated methane reforming across multiple metals;^17^ transfer from reforming to CMD must account for the changed reaction network and gas composition.

Carbon filaments can develop by surface-mediated growth involving steps and restructuring, or through carbon incorporation, transport and precipitation.^18–21^ These routes are not mutually exclusive material labels. Their relative importance varies with particle structure, phase and operating conditions. Systematic DFT studies distinguish surface adsorption, subsurface absorption and migration barriers,^22^ reinforcing the need to separate methane-derived C* supply from subsequent carbon growth. These distinctions define the domain in which a surface carbon-affinity index can inform the activity–carbon relationship.

Here, we retain Fe, Ni, Pt and Cu as the UBI-QEP core set and evaluate the original *D*_C_ formulation quantitatively. The analysis addresses descriptor redundancy, reference-state dependence, external energetic benchmarking, condition-dependent thermodynamics and the interpretation of experimental activity and deactivation. The contribution is the explicit endpoint transformation, an external comparison of energetic ordering, and condition-resolved literature contrasts that define the limitations of transferring elemental energetic rankings to catalyst performance.

## 2 Descriptor development and theoretical framework

### 2.1 Methane dehydrogenation sequence

The sequence is written using * for an available surface site. These formal reactions identify stoichiometric endpoints; they do not impose quasi-equilibrium on the reacting surface.R1
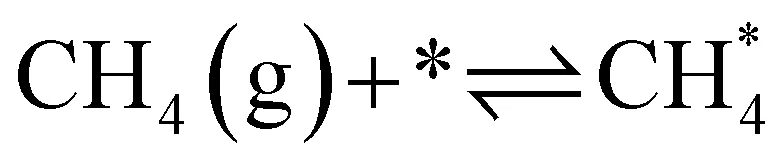
R2-R5
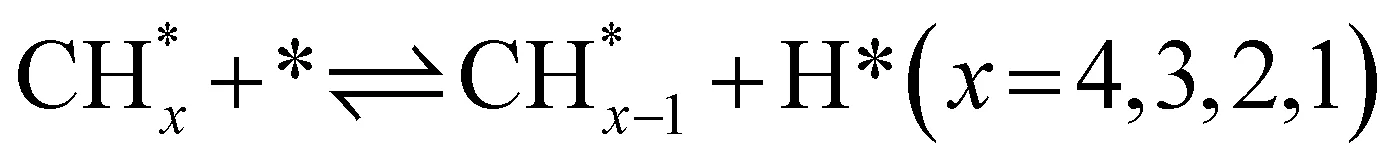

R6 2H* ⇌ H_2_(g) + 2*

The *x* = 1 product is C*. Site occupancies in these equations are formal; real C and CH_*x*_ adsorbates can require multiatom ensembles. Microscopic reverse pathways exist, but their presence does not imply appreciable reverse flux under hydrogen-poor CMD conditions. Neither irreversible reactor operation nor low gas-phase H_2_ alone establishes the reversibility of every elementary step.^23^ A Boltzmann distribution over CH_*x*_ states would require evidence of quasi-equilibrium that is not established here. A steady-state coverage prediction would require explicit forward and reverse rates, site balances and carbon-removal pathways.

### 2.2 UBI-QEP energetic framework

We use positive *Q* values for exothermic adsorption from the corresponding gas-phase atom or fragment. With all quantities expressed in the same energy units, the original framework writes the dehydrogenation enthalpy and its intrinsic barrier estimate as

1 Δ*H*_*x*_ = *Q*(CH_*x*_) + *D*(CH_*x*−1_–H) − *Q*(CH_*x*−1_) − *Q*(H)

2 *E*_a,*x*_ = 1/2{Δ*H*_*x*_ + *Q*(CH_*x*−1_)*Q*(H)/[*Q*(CH_*x*−1_) + *Q*(H)]}

Eqn (2) is the semi-empirical expression used in the original analysis, not a general kinetic law. Its correlation between reaction energy and barrier is built into the construction. UBI-QEP does not resolve the electronic structure, spin state, facet-specific adsorption geometry or transition-state relaxation as explicitly as DFT. The often-cited 1–3 kcal mol^−1^ accuracy^16^ is not adopted as a statistically calibrated error distribution for this particular four-metal parameter set. Its built-in reaction-energy dependence should be distinguished from the more general physical concepts underlying electronic-structure descriptors and activation-energy scaling.^24,25^

### 2.3 Carbon affinity and endpoint dependence

Summing the four dehydrogenation enthalpies cancels all intermediate-fragment terms. For the formal reaction 
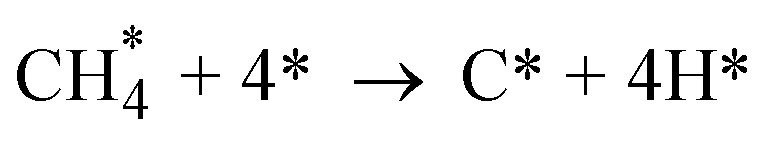
, the result is

3 *D*_C_ = *D*_atom_(CH_4_) + *Q*(CH_4_) − *Q*(C) − 4*Q*(H)

*D*_atom_(CH_4_) denotes complete gas-phase atomization to C(g) + 4H(g), not the first C–H bond dissociation energy. For a common methane adsorption reference, *D*_C_ is exactly an affine transformation of *Q*(C) + 4*Q*(H). Its added content relative to *Q*(C) is the explicit choice of an adsorbed-hydrogen endpoint and associated energy bookkeeping. It contains no new information about the intermediate pathway, nucleation barrier or carbon transport. More negative *D*_C_ means a more favorable specified endpoint; it does not establish a higher steady-state carbon-growth rate.

The endpoint distinction becomes explicit when two hydrogen-recombination events are added and methane is referenced to the gas phase. Within the same chemisorption-enthalpy convention,

4 Δ*H*[CH_4_(g) + * → C* + 2H_2_(g)] = *D*_C_ − *Q*(CH_4_) + 4*Q*(H) − 2*D*(H_2_)

5 = *D*_atom_(CH_4_) − 2*D*(H_2_) − *Q*(C)

Thus, the hydrogen-binding terms cancel. This cancellation is a thermochemical identity, not a kinetic assumption. Hydrogen can still affect CMD strongly through surface coverage, reverse rates and gas chemical potential. *D*_C_ is consequently retained as a semi-empirical carbon-affinity index with an explicit endpoint, rather than an independent universal descriptor. The numerical gas-phase reference check uses NIST thermochemistry^26^ and is detailed in SI Section S4.

### 2.4 Operating conditions and carbon growth

For CH_4_(g) → C(graphite) + 2H_2_(g), ideal gases and unit graphite activity give

6 Δ*G*(*T*,*p*) = Δ*G*°(*T*) + *RT* ln[(*p*_H_2__/*p*°)^2^/(*p*_CH_4__/*p*°)]

This expression describes the overall graphite-referenced reaction. It is not the free energy of forming an isolated C* adsorbate. A quantitative C* phase boundary additionally requires compatible electronic and thermal references, adsorbate free energies, coverage and a surface model. Absolute C* boundary temperatures and critical hydrogen pressures are therefore not inferred here. Surface carbon must still overcome C–C coupling, step-edge attachment or transport barriers before forming a macroscopic carbon phase.^18–22^

## 3 Computational methods

### 3.1 Core data and reproducibility

The Fe, Ni, Pt and Cu chemisorption energies used as input parameters were taken from the published UBI-QEP study of Hei *et al.*,^17^ while the analytic UBI-QEP framework follows Shustorovich and Sellers.^16^ Using these published inputs, the gas-phase C–H dissociation energies and corresponding UBI-QEP reaction energetics were recalculated. The equations, source parameters, intermediate values and calculated results are provided in the SI and accompanying calculation file.

The implied common term *D*_atom_(CH_4_) + *Q*(CH_4_), obtained by rearranging eqn (3), spans 1683.832–1684.536 kJ mol^−1^. This sub-kJ spread is compatible with rounding, but back-calculating it is an internal closure check rather than independent verification of the gas-phase inputs. Relative *D*_C_ differences are fully determined by the stated chemisorption inputs when the common term is held fixed.

### 3.2 External comparisons and statistics

The external energetic comparison of carbon-binding order is extracted directly from Tables S1–S3 of Piqué *et al.*^22^ 14 transition metals spanning 3d, 4d and 5d series, including all four core metals. The common protocol uses VASP/PAW with PBE, 415 eV cutoff, six-layer slabs and isolated atomic C as reference. We select the strongest surface adsorption site on fcc(111), bcc(110) or hcp(0001), excluding subsurface absorption entries. These are crystallographically corresponding dense surfaces, not identical Miller planes. The source treats Fe, Co and Ni spin-polarized and uses low coverages of 1/9 or 1/8 monolayer; its nonmagnetic treatment of Cr is a limitation. Source labels bcc(011) and (110) are symmetry-equivalent. The four overlapping metals alone enter the paired UBI-QEP-DFT statistics; the other ten provide external energetic context. No new DFT calculation or additional UBI-QEP parameter is introduced.

DFT adsorption energy is defined as *E*(slab + C) − *E*(slab) − *E*[C(g)], negative for favorable binding. Signed *E*_ads_ is compared with positive *Q*(C); a negative correlation is therefore expected for consistent ordering. We also show binding magnitudes for visual comparison. Pearson *r*, ordinary-least-squares *R*^2^ with an intercept, and Spearman *ρ* are calculated without statistical significance claims. Here *R*^2^ equals *r*^2^; it is not agreement with the identity line or a predictive validation score. Small sample size and the leverage of the weak-binding Cu endpoint are assessed explicitly in the SI.

Experimental evidence is kept separate from energetic benchmarking. We extract reported numerical contrasts within individual studies and preserve temperature, preparation, time basis and measured quantity. Conversion, hydrogen yield, carbon yield and apparent activation energy are not pooled into one regression. A supported catalyst is not assigned a new measured adsorption energy merely because its principal metal is Fe or Ni. Accordingly, these experiments test the adequacy of a metal-only interpretation rather than validate a universal fitted performance law.

### 3.3 Thermodynamics and uncertainty

The equilibrium calculation uses the tabulated NIST-JANAF methane formation Gibbs energies,^27^ reversed for decomposition to standard graphite and H_2_. This avoids reconstructing the reaction from inconsistent thermal reference conventions. For a pure methane feed at fixed total pressure *P* with no inert gas, the equilibrium extent *X* follows

7 *K*_p_ = exp[−Δ*G*°/(*RT*)] = 4(*P*/*p*°)*X*^2^/(1 − *X*^2^)

8
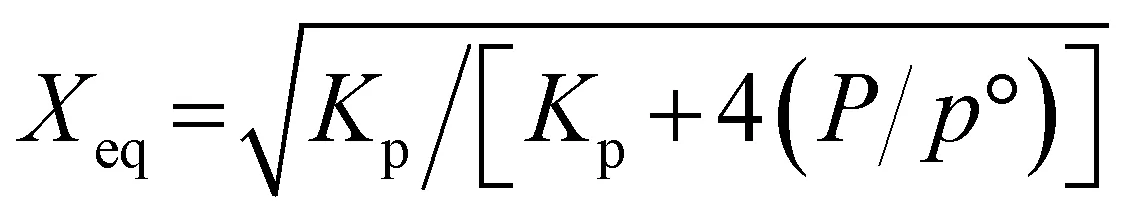


Input sensitivity is recalculated using 200 000 independent samples per scenario and random seed 20 260 920. Additive Gaussian standard deviations of 1, 2 and 3 kcal mol^−1^ are compared with bounded uniform ±10% perturbations and Gaussian 10% standard deviations. These are declared sensitivity scenarios, not measured uncertainty distributions. No uncertainty in an intrinsic barrier is inferred from descriptor uncertainty alone.

## 4 Results and discussion

### 4.1 Thermodynamic window for methane decomposition

Table 2 gives corrected, internally consistent graphite-referenced values. The standard-state sign change occurs near 820 K by interpolation between the 800 and 900 K JANAF entries. It is not a universal onset temperature. The reaction quotient can favor decomposition below this temperature when hydrogen activity is sufficiently low. At Δ*G*° = 0 and *P* = *p*°, eqn (8) gives *X*_eq_ = 1/√5 = 0.4472. Catalysts influence the approach to equilibrium, not this equilibrium composition for a fixed reaction and carbon reference.

**Table 1:** Published UBI-QEP chemisorption energies used as input parameters for the present recalculation. Values are taken from Hei *et al.*;^17^ the UBI-QEP formalism follows Shustorovich and Sellers.^16^

Metal	*Q*(C)	*Q*(CH)	*Q*(CH_2_)	*Q*(CH_3_)	*Q*(H)	*D*_C_
Fe	200.0	142.3	104.4	58.1	63.5	−215
Ni	171.0	116.0	82.6	44.9	63.0	−86
Pt	150.0	97.4	67.6	36.1	61.0	+36
Cu	120.0	71.6	47.5	24.6	56.0	+245

**Table 2:** Recalculated graphite-referenced CMD equilibrium for pure CH_4_ feed at 1 bar. Δ*G*° = −Δ_f_*G*°[CH_4_(g)] from ref. 27

*T*/K	Δ*G*°/kJ mol^−1^	*K*_p_	*X*_eq_
700	+12.643	0.1139	0.1664
800	+2.115	0.7276	0.3923
900	−8.616	3.163	0.6645
1000	−19.492	10.43	0.8501
1100	−30.472	27.99	0.9354
1200	−41.524	64.19	0.9702
1300	−52.626	130.2	0.9850

Increasing hydrogen pressure tenfold at fixed methane pressure raises Δ*G* by 2*RT* ln 10: 30.6, 38.3 and 45.9 kJ mol^−1^ at 800, 1000 and 1200 K, respectively. Increasing methane pressure tenfold at fixed hydrogen pressure lowers Δ*G* by *RT* ln 10. At fixed gas mole fractions, increasing total pressure opposes decomposition because the net gas mole change is +1. These directional thermodynamic predictions do not determine whether filaments or encapsulation will form. Non-graphitic carbon has a different chemical potential; no universal temperature offset is assigned to all carbon morphologies.

### 4.2 CH_*x*_ energetic landscape

The retained CH_*x*_ ladder binds more strongly on Fe than on Cu (Table 1), consistent with the published UBI-QEP input set. Using these published chemisorption inputs, the gas-phase C–H dissociation energies were independently obtained from NIST thermochemical data and used with eqn (2) to recalculate the R3–R5 intrinsic UBI-QEP barriers. The recalculated values agree closely with the published values, with differences below approximately 1.6 kJ mol^−1^ (SI Section S1 and Table S4). The *R*^2^ barrier values are retained directly from the published UBI-QEP dataset because the required methane adsorption energy, *Q*(CH_4_), was not independently available in the retained input set. These intrinsic barriers are used only for energetic consistency and are not treated as experimental apparent activation energies or independent kinetic validation. A largest local barrier cannot identify the rate-controlling step without intermediate populations, reverse fluxes, and a consistent free-energy reference. In particular, low terminal-scission barriers do not by themselves demonstrate rapid carbon production when upstream CH_*x*_ populations are small. We therefore make no mechanistic claim from numerical agreement between an intrinsic barrier and a measured apparent activation energy. Rate-control analysis instead evaluates how perturbations to transition-state and intermediate free energies influence the overall steady-state rate.^28^

### 4.3 Quantitative assessment of descriptor redundancy

The original data give *r*[*D*_C_,*Q*(C)] = −0.993, *R*^2^ = 0.987 and *ρ* = −1.000. For *Q*(C) + 4*Q*(H), the corresponding values are *r* = −1.0, *R*^2^ = 1.0 and *ρ* = −1.0 (Fig. 1). The latter near-exact relationship is required by eqn (3); its small residual results from rounded tabulated quantities. Thus, the formulation is not an independent descriptor beyond established carbon-binding energetics.

**Fig. 1:**
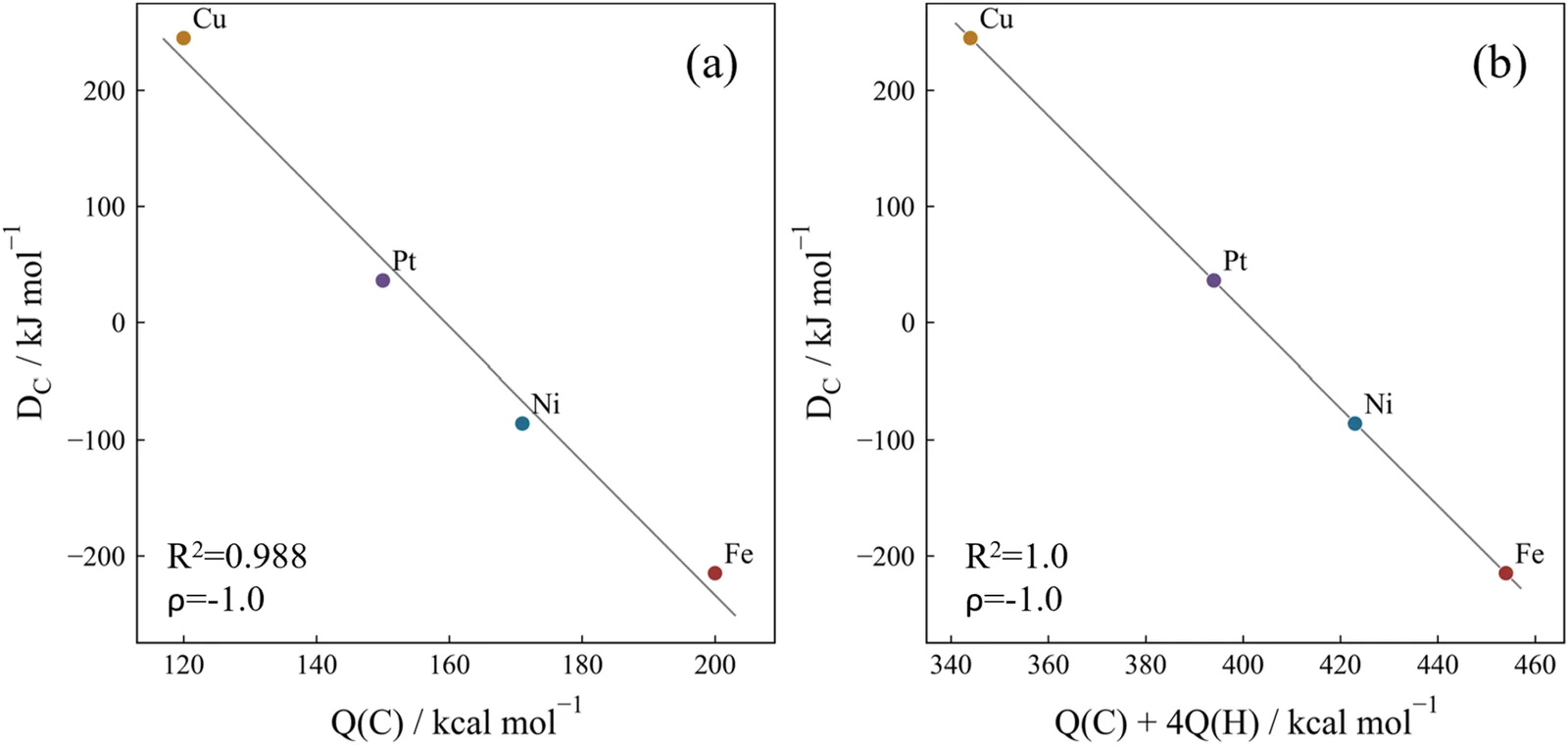
Internal correlations within the original four-metal dataset (Cu, Pt, Ni, and Fe) using (a) carbon binding energy, *Q*(C), and (b) the combined binding-energy descriptor, *Q*(C) + 4*Q*(H). Solid lines are descriptive least-squares fits with fitted intercepts.

*D*_C_ can nevertheless be retained transparently as a reaction-energy index. Relative to Fe, its reported increments are 129, 251 and 460 kJ mol^−1^ for Ni, Pt and Cu. Of the Fe-to-Cu separation, approximately 335 kJ mol^−1^ derives from *Q*(C) and 126 kJ mol^−1^ from 4*Q*(H), before rounding. Hydrogen binding therefore contributes about 27.3% of this particular separation, even though the rank is identical. This illustrates what the complete adsorbed-H endpoint adds numerically without implying a new degree of independent physical information. For an H_2_ gas endpoint, that contribution cancels exactly as shown in eqn (4) and (5).

### 4.4 External DFT energetic comparison and experimental contrasts

For Fe, Ni, Pt and Cu, the verified PBE carbon-binding magnitudes are 894, 651, 707 and 440 kJ mol^−1^, respectively.^22^*Q*(C) *versus* signed *E*_ads_ gives *r* = −0.929, *R*^2^ = 0.862 and *ρ* = −0.800; using binding magnitudes reverses the two correlation signs. The semi-empirical order Fe > Ni > Pt > Cu becomes Fe > Pt > Ni > Cu in DFT (Fig. 2). Five of six pairwise orders agree, but the Ni–Pt inversion precludes treating the original rank as universal. Excluding Cu gives magnitude *r* = 0.794 for only three observations. Neither coefficient establishes predictive accuracy or enlarges the paired sample beyond four.

**Fig. 2:**
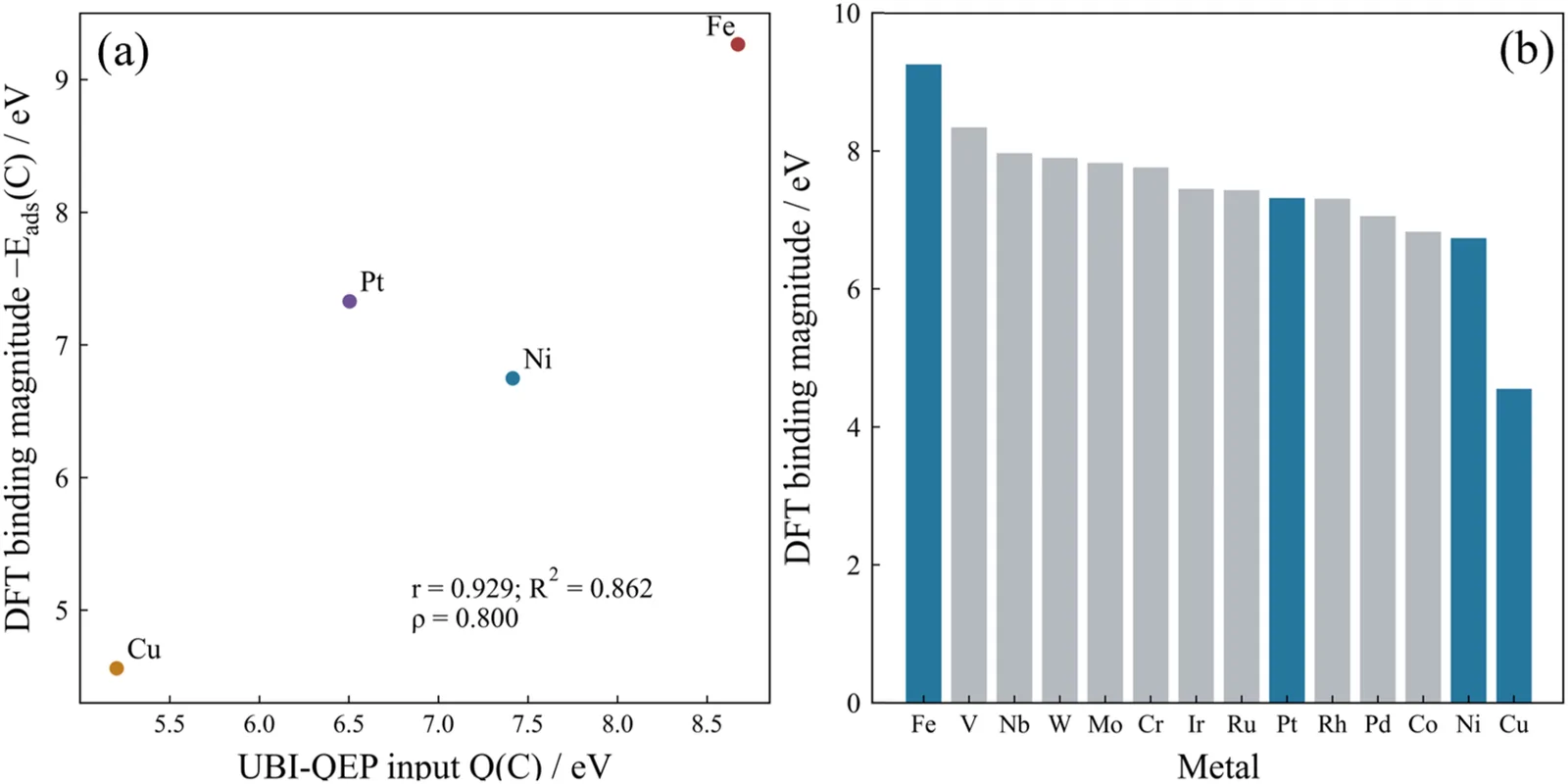
External DFT comparison of carbon-binding order using the dataset of Piqué *et al.*^22^ (a) Four paired UBI-QEP inputs and PBE binding magnitudes. (b) Fourteen metals evaluated using the same DFT protocol on the specified dense facets; blue identifies the four core metals.

Facet sensitivity is already visible within this one DFT source. Ni(001) and Pt(001) bind C by 792 and 751 kJ mol^−1^, reversing the Ni(111)/Pt(111) ordering. Across the three reported low-index facets, Fe, Ni, Pt and Cu span 748–894, 651–792, 662–751 and 440–584 kJ mol^−1^, respectively. These ranges describe structural variation, not statistical error bars. The original empirical inputs lack equally explicit facet and thermal conventions; the comparison therefore tests ordering and transferability rather than absolute method accuracy.

A method-dependent inversion can arise from the original empirical parameterization, facet and adsorption-site specificity, magnetic treatment or electronic-structure approximations. The available comparison cannot partition these contributions. The weaker Cu endpoint is comparatively consistent, but the Fe/Ni/Pt ordering should not be promoted to a universal carbon-affinity sequence. Nor can Pt carbon tolerance be justified simply by assigning it weaker atomic C binding than Fe and Ni. Atomic C adsorption, graphitic-nucleus stability and sustained carbon accumulation are different observables.

The within-study experimental contrasts in Fig. 4 illustrate the limitations of a metal-only activity–deactivation interpretation. In a matched Fe/Al_2_O_3_ study, catalysts with the same nominal metal loading and calcination temperature showed initial methane conversions of 20.8% and 47.8% for co-precipitation and impregnation, respectively.^29^ A metal-only descriptor assigns them the same value and cannot explain this 27.0 percentage-point difference. NiCu/C also exhibited apparent activation energies of 86 and 45 kJ mol^−1^ in different temperature regimes, with accompanying changes in carbon-fiber structure.^30^ These observations identify missing state and transport information; they are not pointing on a universal cross-catalyst activity fit.

A second matched comparison concerns FeCo catalysts.^31^ At 700 °C, maximum CH_4_ conversions for nominal Fe : Co : Al ratios 1 : 1 : 1, 2 : 1 : 1 and 3 : 1 : 1 were 81.1, 83.0 and 78.1% after *in situ* activation, *versus* 26.1, 42.0 and 36.7% after prior H_2_ reduction. These 55.0, 41.0 and 41.4 percentage-point differences occur within matched composition pairs. Conversion retention after 650 min was also reported, providing activity and time-on-stream evidence beyond a single initial point (SI Table S11). Precursor evolution and catalyst mass basis prevent interpreting these results as intrinsic turnover frequencies (Table 3).

**Table 3:** Independent within-study literature contrasts and their intended interpretation. Reported quantities are retained in their original measurement categories and are not combined into a harmonized activity or validation database. Initial and maximum CH_4_ conversion, retained fraction, H_2_ yield, and apparent activation energy are treated as distinct observables

System and source	Conditions or basis	Reported observation	Implication
20 wt% Fe/Al_2_O_3_ (ref. 29)	700 °C; 400 °C calcination; initial values	CH_4_ conversion 20.8% (CP) and 47.8% (Imp)	Preparation changes performance at fixed metal identity
Fe/Al_2_O_3_ Imp^29^	800 °C; 120 min run	≈80–85% conversion; reactor plugging ended run	Sustained activity can coexist with process failure
NiCu/C^30^	450–600 °C; two fitted regimes	*E*_app_ = 86 and 45 kJ mol^−1^	Kinetic regime changes at fixed catalyst family
NiCu/C^32^	500 °C; 15–25% Cu compositions	Initial growth comparable to Ni; more sustained growth	Cu need not always reduce initial activity
Ni/SiO_2_ microflakes^33^	Maximum reported yield; study-specific test	H_2_ yield 74%; slight deactivation	Yield is not a site-normalized rate
Supported Fe, Co and Pd^34–36^	Separate CMD studies; conditions differ	Filament/CNF/CNT formation reported	Qualitative capability only; no common rank
FeCo from LDH^31^	700 °C; 650 min; three matched compositions	Maximum conversion 78.1–83.0% *in situ*; 26.1–42.0% pre-reduced	Activation history changes conversion at fixed nominal composition

The common observation that Ni can outperform Fe is therefore not resolved by insisting that the lowest initial barrier must give the highest turnover. Surface coverage, the fraction of accessible ensembles, reduction and carburization, particle size and carbon removal can all change the effective rate. In the Fe/Al_2_O_3_ comparison, preparation-dependent behavior is observed directly;^29^ assigning a unique carbide or diffusion explanation without *operando* evidence would go beyond those data. The stronger general conclusion is that the original energetic ranking is insufficient, not that one particular omitted process has been proved responsible. Independent Fe CMD studies report strong sensitivity to catalyst preparation and composition,^37,38^ while Co promotion introduces an additional compositional variable.^39^

### 4.5 Carbon formation and catalyst design implications

For surface-mediated growth, carbon diffusion and C–C coupling must generate a stable nucleus. Step geometry and graphene–metal edge matching can stabilize nuclei and alter the required nucleus size.^18–20^ Such growth can produce filaments without requiring the carbon flux to cross the particle bulk. Conversely, surface growth can encapsulate a particle if graphitic coverage spreads over the methane-activation ensembles. Neither outcome follows from isolated-atom binding alone. Atomistic nucleation and nanotube-growth studies likewise require collective carbon configurations and evolving particle structure.^40–42^

For dissolution–precipitation, carbon enters subsurface or bulk sites, migrates under a chemical-potential gradient and precipitates at a growth interface.^21,43,44^ Ni and Fe can participate in this route; Co and Pd also have direct filament-growth evidence.^34–36^ Carbon permeability is a phase-, size- and temperature-dependent property, not a binary label inferred from *Q*(C). For Cu, low bulk accommodation and weak activation make it an inappropriate analogue of conventional Ni filament catalysts, but they do not imply that all surface carbon growth on Cu is impossible. Pt likewise should not be classified as universally carbon-impermeable.^22^ Historical nickel studies and carbon-fiber analyses distinguish nucleation, transport and deposition.^45–47^ The acetylene feed in the first study is mechanistic context, not direct CMD validation; bulk-solubility calculations provide complementary information to surface adsorption.^48^

The same DFT source resolves the additional physical steps quantitatively (Table 4). Ni(111) and Cu(111) have lower surface-migration than entry barriers, whereas Pt(111) shows the opposite comparison for the specified paths. Thus neither surface binding nor a binary “carbon-permeable” label specifies the dominant carbon flux. Different paths connect particular local minima, so rate ratios also require occupancies and prefactors. Subsurface hopping is not a measurement of bulk permeability, and fast migration on Cu does not establish sufficient methane activation or a stable filament nucleus.

**Table 4:** Published atomic-C migration barriers on the core dense surfaces, kJ mol^−1^,^22^ SI Table S10. (These are elementary diffusion barriers; they are not measured filament-growth barriers)

Surface	Surface migration	Subsurface migration	Surface → subsurface	Emergence
Fe(110)	73.4	55.0	131.1	85.9
Ni(111)	36.2	116.7	64.5	6.6
Pt(111)	80.2	75.7	39.4	38.0
Cu(111)	8.4	89.7	50.7	7.9

The two routes can be written as competing carbon fluxes from a shared C* population. A schematic balance is *r*_form_ − *r*_surface-growth_ − *r*_entry_ + *r*_emergence_ − *r*_hydrogenation_ = dNC*/d*t*. Predicting their partition requires rate constants, adsorption ensembles, particle geometry and incorporation/nucleation energetics. No filament probability is calculated here because the required metal- and condition-specific quantities are absent. Fe additionally undergoes phase evolution and carburization; a bulk carbide formation energy alone does not establish its *operando* abundance or activity. These effects are incorporated as physical limits on the original carbon-affinity interpretation, not as an arbitrarily weighted composite descriptor.

Temperature simultaneously changes methane activation, diffusion, nucleation, sintering and equilibrium (Fig. 3). Their competition prevents a universal prediction that higher temperature improves or worsens lifetime. Higher H_2_ pressure lowers the graphite-forming driving force at fixed CH_4_ pressure, while also changing adsorption and hydrogenation rates. Increased methane supply can accelerate carbon accumulation when transport or nucleation cannot keep pace. These are directional implications with specified constraints; morphology transitions require an independently constrained kinetic model. Consistent with this distinction, supported Co/Ni experiments at 773 K identify separate gas-composition thresholds for carbon formation and deactivation.^49^ Their existence supports a condition-dependent filament-growth window; the threshold values are catalyst-specific, not functions of *D*_C_ alone.

**Fig. 3:**
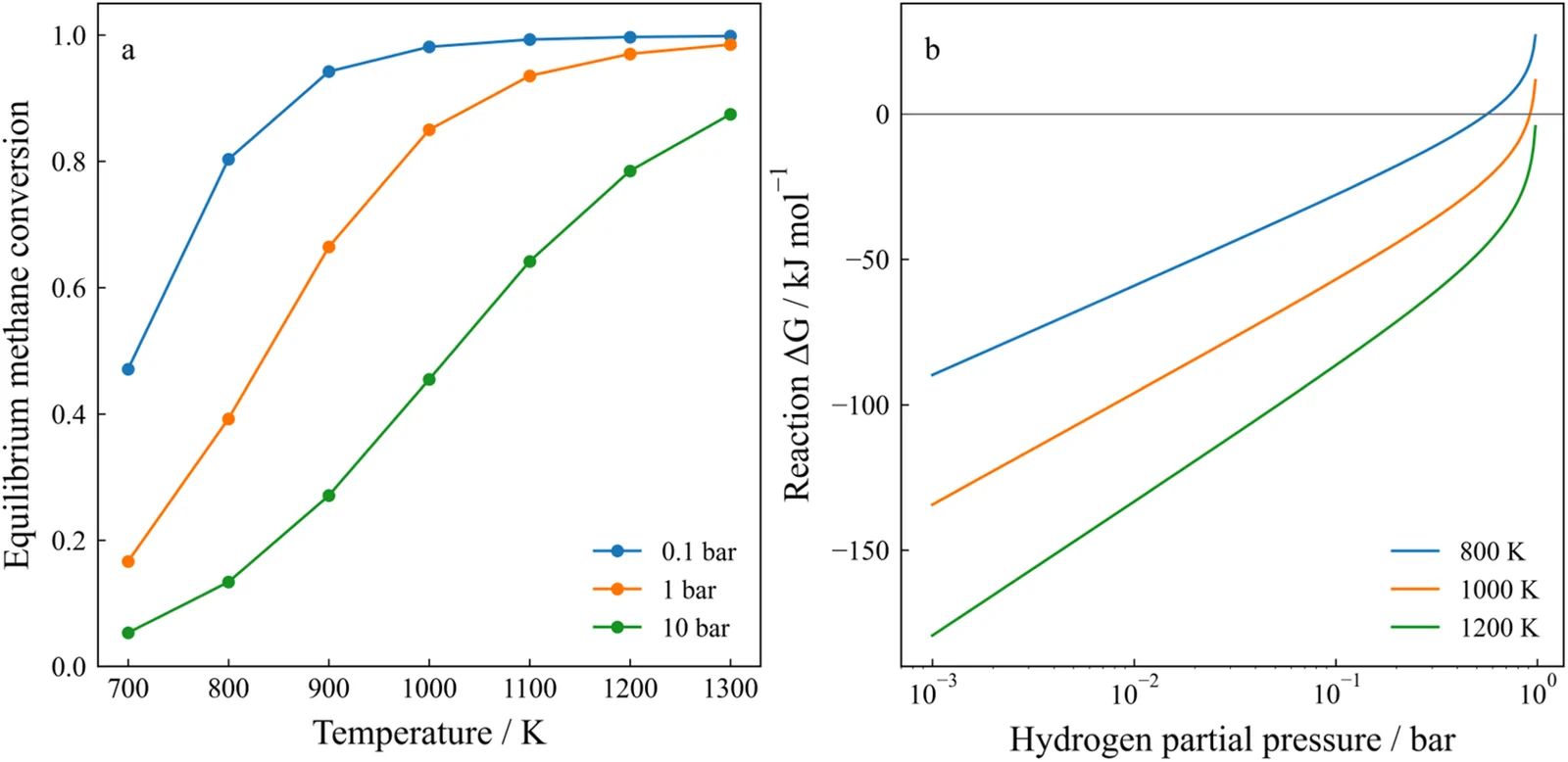
Quantitative operating-condition effects for graphite-referenced CMD. Panel (a) uses NIST-JANAF values at the displayed temperatures and eqn (8), with a pure methane feed and fixed total pressure. Panel (b) uses eqn (6) at a total CH_4_/H_2_ pressure of 1 bar.

**Fig. 4:**
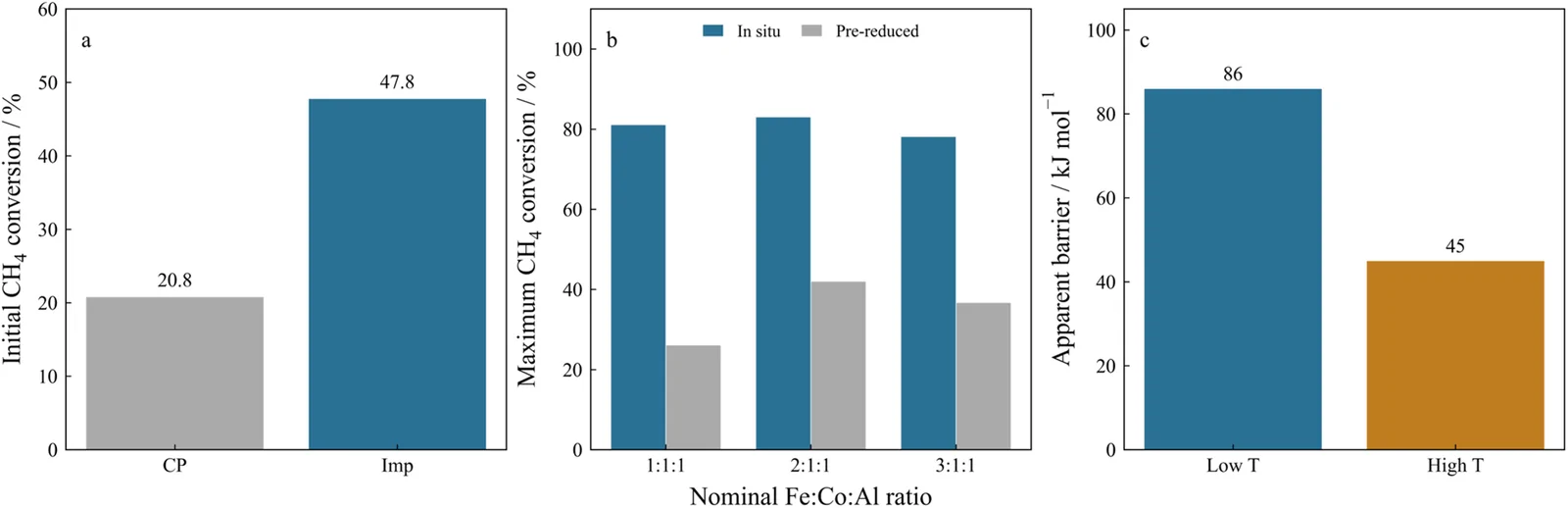
Quantitative within-study experimental contrasts. (a) Initial CH_4_ conversion for 20 wt% Fe/Al_2_O_3_ prepared by co-precipitation (CP) and impregnation (Imp), calcined at 400 °C and tested at 700 °C.^29^ (b) Maximum CH_4_ conversion during 650 min tests at 700 °C for *in situ* activated and pre-reduced FeCo catalysts; the plotted values are maximum conversions and not the retained fractions after 650 min.^31^ (c) Apparent activation energies for the two reported temperature regimes of NiCu/C.^30^

Ni–Cu illustrates why the original design argument must be qualified. Moderate Cu addition can prolong growth while retaining initial activity, whereas larger additions can reduce carbon supply and growth.^32^ Changes in ensembles, solubility and transport accompany any change in binding energy. A linear interpolation between elemental *D*_C_ values is therefore not an alloy model. Likewise, more carbon does not necessarily mean faster deactivation: accessible filament growth can continue, while bed plugging or mechanical damage can ultimately limit operation.^29,50^ This interpretation is consistent with earlier Ni–Cu carbon-capacity, preparation and crystallite-size studies.^51–53^

### 4.6 Robustness and limitations

For independent equal input standard deviations *σ* in *Q*(C) and *Q*(H), eqn (3) gives *σ*(*D*_C_) = √17 *σ* when the common reference term is fixed. Thus *σ* = 2 kcal mol^−1^ gives 34.5 kJ mol^−1^. The recalculated complete-order retention probabilities are 100.0%, 98.9% and 91.1% for Gaussian input standard deviations of 1, 2 and 3 kcal mol^−1^, respectively. Bounded uniform ±10% perturbations preserve the order in 75.4% of samples; Gaussian 10% standard deviations preserve it in 45.9%. The distinction is substantial and the distributions are not interchangeable.

These probabilities are conditional input-sensitivity results. Common reference errors shift all *D*_C_ values without changing their order, whereas correlated or metal-specific errors can alter separations. UBI-QEP model discrepancy, source selection and DFT facet or functional differences are not captured by resampling atomic inputs. The verified Ni–Pt inversion and facet dependence in Fig. 2 already show why within-model robustness cannot establish external accuracy. The distinction between input spread and model discrepancy also motivates uncertainty-aware density-functional methods;^54^ no such calibrated ensemble is available for the present legacy parameter set.

Coverage-dependent interactions and site blocking can change both binding and activation. Hydrogen coverage depends on adsorption/desorption kinetics as well as partial pressure; carbon can reconstruct steps, change the active phase or remove access to the surface. Particle-size and support effects modify the number and identity of active ensembles.^55^ Modern high-throughput DFT and machine learning can explore these variables, but their predictions remain conditional on their training structures and target observables.^22,56^ They establish an appropriate comparison standard, not automatic validation of the present index. Ni support and structural evolution have been examined directly.^57–60^ A particle-size study spanning 13.2–25.4 nm associates larger Ni particles with nanotubes and smaller ones with encapsulation under its conditions;^61^ it does not establish a universal critical particle size.

Modern methane-cracking screening targets specific elementary barriers and alloy environments.^62^ Sun *et al.*'s experimental screening includes mechanically driven catalysts,^56^ so its performance cannot be transferred directly to a purely thermal CMD ranking. Open reaction databases can improve provenance and reproducibility,^63^ but only when facet, composition, coverage and energy references are retained.

The present framework supports transparent comparisons of specified thermochemical endpoints and explicit tests of energetic ordering. It does not yet provide a calibrated performance predictor. The external compilation broadens the discussion but does not solve the limited paired sample size or substitute for a harmonized experimental database. A defensible next validation would match catalyst phase, particle size, support, feed, temperature and time-on-stream, then compare measured rates and carbon morphology with independently calculated surface energetics and transport barriers. Repeated measurements of one metal would not create additional independent elemental descriptor values. Fixed- and fluidized-bed results also require separate residence-time and transport accounting.^64,65^ The classical distinction between carbon accumulation and loss of accessible sites remains central.^66,67^

## 5 Conclusions

The present analysis refines the physical interpretation and predictive scope of the UBI-QEP carbon-affinity quantity. *D*_C_ remains useful as a transparent, integrated reaction enthalpy for the specified methane-to-C* endpoint. However, its near-perfect correlation with *Q*(C) + 4*Q*(H) arises from its mathematical construction and does not establish an independent universal descriptor. When the thermochemical cycle is closed using gaseous H_2_ as the hydrogen endpoint, the hydrogen-binding contribution cancels, revealing the close relationship between *D*_C_ and conventional carbon-binding energetics. Comparison with a consistent 14 metal PBE dataset supports the broad energetic trend but reveals a Ni–Pt rank inversion among the four paired metals, demonstrating the sensitivity of catalyst rankings to computational method, surface structure, and reference state. Experimental comparisons further show that elemental carbon affinity alone cannot predict methane-conversion activity, carbon morphology, or catalyst deactivation. No out-of-sample model for catalytic activity or catalyst lifetime is developed or validated in the present work. Graphite referenced equilibrium calculations quantify the effects of temperature and pressure without imposing quasi-equilibrium on adsorbed CH_*x*_ intermediates. Overall, carbon affinity provides one energetic component of catalytic methane decomposition, while sustained activity and the formation of filamentous or encapsulating carbon additionally depend on carbon transport, nucleation, surface structure, and catalyst-phase evolution. The main contribution of this work is therefore a reference-state-consistent interpretation of carbon affinity and a quantitative definition of the conditions under which elemental energetic rankings can, and cannot, be transferred to practical catalyst performance.

## Conflicts of interest

There are no conflicts to declare.

## Supplementary Material

RA-OLF-D6RA05232K-s001

## Data Availability

The data supporting this study, including the UBI-QEP inputs, recalculated reaction barriers, correlation statistics, equilibrium calculations, and numerical literature comparisons, are provided in the supplementary information (SI) file. Literature-derived data are attributed to their original sources in the manuscript and SI. Supplementary information is available. See DOI: https://doi.org/10.1039/d6ra05232k.
