# Supplementary material for: Evaluating carbon affinity in catalytic methane decomposition using UBI-QEP energetics and DFT carbon-binding analysis
Source: RSC Adv. 2026 Oct 20;16(48):64669–79. doi: 10.1039/d6ra05232k (PMC13681386; doi:10.1039/d6ra05232k)
Supplement: Supplementary file 1 [file RA-016-D6RA05232K-s001.pdf]

Supplementary Information (SI) for RSC Advances.
This journal is © The Royal Society of Chemistry

Supporting information

# Evaluating Carbon Affinity in Catalytic Methane Decomposition Using UBI-QEP Energetics and DFT Carbon-Binding Analysis

Yahya Sorkhe[1,2]

[1]Department of Metallurgical and Chemical Engineering, Istanbul Technical University, Istanbul 34469, Turkey.

[2] Faculty of science, Koc University, Istanbul, Turkey.

Corresponding author: Sorkhe20@itu.edu.tr

This Supporting Information provides the provenance and recalculation of the UBI-QEP dataset, independently recalculated statistics, verified external DFT inputs, thermodynamic calculations, and condition-resolved experimental evidence.

## S1. Provenance and recalculation of the UBI-QEP dataset.

The UBI-QEP analysis used in this work distinguishes between published chemisorption input parameters and quantities recalculated in the present study. The chemisorption energies Q(C), Q(H), Q(CH), Q($CH_2$), and Q($CH_3$) for Fe, Ni, Pt, and Cu were taken from the published UBI-QEP treatment of methane-related surface chemistry. Using these published chemisorption inputs, the gas-phase C–H dissociation energies were independently recalculated from standard gas-phase enthalpies of formation obtained from the NIST Chemistry WebBook. These gas-phase quantities were then used together with the UBI-QEP expression to recalculate the intrinsic reaction barriers for the successive $CH_x^*$ □$CH_{x-1}^*$ + H* steps. The recalculated R3–R5 barriers closely reproduce the corresponding published values. Small differences arise from rounding of the historical chemisorption parameters and the use of independently tabulated gas-phase thermochemical data. The R2 step additionally requires the methane adsorption energy Q($CH_4$), which is not independently specified in the retained input table. Table S1 lists the published chemisorption inputs and their provenance. Table S2 provides the gas-phase thermochemical quantities used in the recalculation. Table S3 compares the recalculated UBI-QEP barriers with the published values.

Table S1. Provenance of the numerical quantities used in the UBI-QEP analysis.

| Quantity | Metals / system | Origin | Use in this work |
|---|---|---|---|
| Q(C), Q(H), Q(CH), Q(CH2), Q(CH3) | Fe, Ni, Pt, Cu | Hei et al. [17] | Published input parameters |

| | | | |
|---|---|---|---|
| UBI-QEP analytic formulation | General | Shustorovich and Sellers [16] | Barrier and reaction-energy equations |
| Gas-phase ΔfH° values | $CH_4$, $CH_3$, $CH_2$, CH, C(g), H(g) | NIST Chemistry WebBook | Calculation of C-H dissociation energies |
| R3-R5 intrinsic barriers | Fe, Ni, Pt, Cu | Recalculated in this work | Compared with published UBI-QEP values |
| R2 intrinsic barriers | Fe, Ni, Pt, Cu | Hei et al. [17] | Retained because $Q(CH_4)$ is not independently available in the retained input set |
| DC and descriptor correlations | Fe, Ni, Pt, Cu | Recalculated / reanalysed in this work | Descriptor analysis |
| DFT C adsorption | 14 metals | Piqué et al. [22] | External energetic comparison |
| Migration barriers | Fe, Ni, Pt, Cu | Piqué et al. [22] | Transport comparison |

Table S2. Published UBI-QEP chemisorption inputs used in the present recalculation. Positive Q values denotes exothermic chemisorption. Values are used as fixed literature inputs; no refitting or modification was performed. (Units: kcal mol−1).

| **Metal** | **Q(C)** | **Q(CH)** | **Q(CH2)** | **Q(CH3)** | **Q(H)** |
|---|---|---|---|---|---|
| Fe | 200.0 | 142.3 | 104.4 | 58.1 | 63.5 |
| Ni | 171.0 | 116.0 | 82.6 | 44.9 | 63.0 |
| Pt | 150.0 | 97.4 | 67.6 | 36.1 | 61.0 |
| Cu | 120.0 | 71.6 | 47.5 | 24.6 | 56.0 |

## S1.1 Gas-phase C–H dissociation energies

The gas-phase bond dissociation energy for each successive methane dehydrogenation step was calculated from standard gas-phase enthalpies of formation according to

$$D_{gas}(CH_x\text{-}H) = ∆_f H^{\circ}(CH_{x-1}) + □_f H^{\circ}(H) - □_f H^{\circ}(CH_x)$$

Standard gas-phase enthalpies of formation were taken from the NIST Chemistry WebBook at approximately 298 K.

Table S3. Gas-phase C-H dissociation energies used in the UBI-QEP recalculation.

| **Step** | **Gas-phase reaction** | **Dgas / kcal mol−1** |
|---|---|---|
| R2 | $CH_4$ → $CH_3$ + H | 104.82 |
| R3 | $CH_3$ → $CH_2$ + H | 109.63 |
| R4 | $CH_2$ → CH + H | 101.75 |
| R5 | CH → C + H | 81.39 |

### S1.2 Recalculation of intrinsic UBI-QEP barriers

For the formal surface dissociation

$$CH_x^* \square CH_{x\text{-}a}^* + H^*$$

The intrinsic barrier was recalculated using:

$$E_a = \frac{1}{2}[D_{gas} + Q_{reactant} - Q_{product} - Q(H) + \frac{Q_{product}Q(H)}{Q_{product} + Q(H)}$$

Here, $Q_{reactant}$ and $Q_{product}$ are the published UBI-QEP chemisorption energies listed in Table S2, and $D_{gas}$ is the corresponding gas-phase C–H dissociation energy from Table S3. For example, for the Fe R3 surface dissociation step, *CH□* □ CH₂* + H**, the published UBI-QEP chemisorption energies are Q(CH₃) = 58.1 kcal mol⁻$^1$, Q(CH₂) = 104.4 kcal mol⁻$^1$, and Q(H) = 63.5 kcal mol⁻$^1$. The corresponding gas-phase C–H dissociation energy is 109.63 kcal mol⁻$^1$. Substitution of these values into the UBI-QEP barrier expression gives: Ea = 19.66 kcal mol⁻$^1$ = 82.25 kJ mol⁻$^1$. The published value for this step is 83 kJ mol⁻$^1$, showing close agreement with the recalculated result. The small difference is consistent with rounding of the historical input parameters and the use of independently tabulated gas-phase thermochemical data.

Table S4. Comparison of recalculated and published UBI-QEP intrinsic barriers.

| **Metal** | **Step** | **Recalculated / kJ mol−1** | **Published / kJ mol−1** | **Difference / kJ mol−1** |
|---|---|---|---|---|
| Fe | R3 | 82.25 | 83 | −0.75 |
| Fe | R4 | 92.59 | 92 | +0.59 |
| Fe | R5 | 17.55 | 17 | +0.55 |
| Ni | R3 | 93.45 | 95 | −1.55 |
| Ni | R4 | 96.61 | 96 | +0.61 |
| Ni | R5 | 19.73 | 19 | +0.73 |
| Pt | R3 | 102.92 | 104 | −1.08 |

| | | | | |
|---|---|---|---|---|
| Pt | R4 | 101.38 | 100 | +1.38 |
| Pt | R5 | 23.34 | 23 | +0.34 |
| Cu | R3 | 118.06 | 119 | −0.94 |
| Cu | R4 | 111.04 | 110 | +1.04 |
| Cu | R5 | 31.74 | 31 | +0.74 |

The small deviations, all within approximately 1.6 kJ mol$^{-1}$, are consistent with rounding of the historical chemisorption parameters and differences in the tabulated gas-phase thermochemical values. These comparisons confirm that the published UBI-QEP barrier sequence can be reproduced from the stated input data and analytic expression.

## S2. Descriptor identity and statistics

Σ ΔHₓ = Q(CH₄) - Q(C) + $D_{atom}$(CH₄) - 4Q(H) (S1)

All Q(CH₃), Q(CH₂) and Q(CH) terms cancel in the sum. The result follows from Hess's law without quasi-equilibrium. The reported $D_C$ values and the combined atomic inputs give the following closure check.

Table S5. The common-term mean is 1684.290 kJ mol⁻$^{1}$ and its range is 0.704 kJ mol⁻$^{1}$. This term is back-calculated, not independently measured. Relative input-based differences from Fe are 129.704, 251.040 and 460.240 kJ mol⁻$^{1}$; reported rounded differences are 129, 251 and 460 kJ mol⁻$^{1}$.

| **Metal** | **Q(C)+4Q(H) / kcal mol⁻$^{1}$** | **Inferred common term / kJ mol⁻$^{1}$** |
|---|---|---|
| Fe | 454.0 | 1684.536 |
| Ni | 423.0 | 1683.832 |
| Pt | 394.0 | 1684.496 |
| Cu | 344.0 | 1684.296 |

Table S6. Pearson coefficients use unweighted pairs; Spearman coefficients use their ranks. $R^2$ is $r^2$ for ordinary least squares with an intercept, not agreement with the identity line. No significance or out-of-sample prediction claim is made. Descriptor correlations are algebraically dependent; the DFT inputs are externally sourced.

| Comparison | n | Pearson r | OLS $R^2$ | Spearman ρ |
|---|---|---|---|---|
| **$D_C$ versus Q(C)** | 4 | -0.993979047 | 0.987994346 | -1.000 |
| **$D_C$ versus Q(C)+4Q(H)** | 4 | -0.999998639 | 0.999997279 | -1.000 |
| Q(C) versus signed DFT Eads | 4 | -0.928517985 | 0.862145649 | -0.800 |

## S3. Endpoint and gas-phase thermochemical reference

Twice adding 2H* → H₂(g) + 2*, with enthalpy 2Q(H) – D(H₂), and referencing methane to the gas phase gives $D_{atom}$(CH₄) – 2D(H₂) – Q(C). Hydrogen adsorption therefore cancels exactly from that endpoint, while hydrogen still influences kinetics and gas chemical potential.

NIST formation enthalpies near 298 K [26] give 716.67, 218.00 and −74.873 kJ mol⁻$^1$ for C(g), H(g) and CH₄(g), respectively. Thus $D_{atom}$(CH₄) = 1663.543 kJ mol⁻$^1$ and D(H₂) = 436.00 kJ mol⁻$^1$. Combining these with the mean original common term would imply Q(CH₄) ≈ 20.75 kJ mol⁻$^1$. This inferred value is not substituted for an independently sourced methane adsorption parameter.

An absolute C* free-energy boundary would require consistent electronic energies, zero-point and vibrational terms, adsorbate coverage and reference-state conversion. Graphite equilibrium is calculated separately; gas-atom entropy is not assigned directly to an adsorbed atom.

## S4. Verified 14-metal DFT benchmark

Source: Piqué et al. [22], supporting file cs2c01562_si_001.pdf, retrieved through the public Europe PMC supplementary-files service for PMC9880994. Tables S1–S3 were checked as PDF tables and extracted text. Their positive adsorption binding convention is converted here to Eads = E(slab+C) – E(slab) – E(Catom). Select the largest surface Eads in the source for the stated facet; never select a subsurface Eabs instead.

Table S7. Surface binding magnitudes B and signed adsorption energies. Conversion: 1 eV per particle = 96.48533212 kJ mol⁻$^1$.

| Metal | Facet | Selected surface site | B / kJ mol⁻$^1$ | Eads / eV |
|---|---|---|---|---|
| V | bcc(110) | hollow | 806 | -8.353601 |

| | | | | |
|---|---|---|---|---|
| Cr | bcc(110) | hollow | 750 | -7.773202 |
| Fe | bcc(110) | hollow | 894 | -9.265657 |
| Co | hcp(0001) | hollow | 660 | -6.840418 |
| Ni | fcc(111) | hcp hollow | 651 | -6.747140 |
| Cu | fcc(111) | fcc hollow | 440 | -4.560279 |
| Nb | bcc(110) | hollow | 770 | -7.980488 |
| Mo | bcc(110) | hollow | 756 | -7.835388 |
| Ru | hcp(0001) | hollow | 718 | -7.441546 |
| Rh | fcc(111) | hcp hollow | 706 | -7.317174 |
| Pd | fcc(111) | hcp hollow | 682 | -7.068432 |
| W | bcc(110) | hollow | 763 | -7.907938 |
| Ir | fcc(111) | hcp hollow | 720 | -7.462274 |
| Pt | fcc(111) | fcc hollow | 707 | -7.327539 |

Protocol: VASP, PAW, PBE; 415 eV plane-wave cutoff; six layers, lower three fixed; 3 × 3 × 1 k-point mesh; dilute C coverages 1/9 or 1/8 monolayer; isolated atom reference. Fe, Co and Ni are spin-polarized; Cr is not. Vacuum gaps and slab cell geometry differ as specified in the primary methods. These choices constitute one consistent computational study but do not remove facet, coverage or magnetic-model limitations. This PBE table is not mixed numerically with the revised-PBE calculations of Pototschnig et al.

Table S8. Core comparison and facet variation. Facet ranges are structural ranges, not calibrated uncertainties.

| Metal | Q(C) / kJ mol⁻1 | Dense-facet B / kJ mol⁻1 | B range over three facets |
|---|---|---|---|
| Fe | 836.800 | 894 | 748–894 |
| Ni | 715.464 | 651 | 651–792 |
| Pt | 627.600 | 707 | 662–751 |
| Cu | 502.080 | 440 | 440–584 |

Binding-magnitude r = 0.928517985, $R^2$ = 0.862145648 and ρ = 0.800. Removing Cu gives r = 0.794057735. Five of six pairwise orders agree; Ni/Pt is the inversion. The fourteen-metal context does not increase the number of paired empirical inputs beyond four. No elemental UBI-QEP parameters are inferred from DFT.

Manuscript Table 4 reproduces the four dense-facet rows from source Table S10. Path labels are Fe: b6/b7/b8/b9; Ni: a13/a14/a17/a19; Pt and Cu: a12/a15 (Pt) or a14 (Cu)/a17/a19 for surface, subsurface, entry and emergence, respectively. Different local-minimum paths should not be collapsed into one equilibrium two-state model. No filament probabilities are derived.

## S5. Temperature, pressure and equilibrium

Table S9. The NIST-JANAF standard pressure is 0.1 MPa = 1 bar. Methane formation is (graphite)+2H₂(g)→CH₄(g); decomposition ΔG° has the opposite sign [27].

| T / K | ΔG° / kJ mol⁻¹ | Kp | Xeq, 1 bar |
|---|---|---|---|
| 700 | +12.643 | 0.113916 | 0.166405 |
| 800 | +2.115 | 0.727625 | 0.392313 |
| 900 | -8.616 | 3.162641 | 0.664490 |
| 1000 | -19.492 | 10.426480 | 0.850137 |
| 1100 | -30.472 | 27.987638 | 0.935389 |
| 1200 | -41.524 | 64.188521 | 0.970226 |
| 1300 | -52.626 | 130.165923 | 0.984980 |

For one mole initial CH₄, gas amounts are 1−X methane and 2X hydrogen, giving yCH₄=(1−X)/(1+X) and yH₂=2X/(1+X). At fixed total pressure, $K_p=4(P/p^\circ)X^2/(1-X^2)$. This calculation excludes initial hydrogen and inert gas. The zero of linearly interpolated ΔG° is approximately 819.7 K, and Xeq at Kp=1 and P=p° is 0.447214. Neither result is a kinetic onset criterion.

## S6. Uncertainty and sensitivity

$$\mathrm{Var}(D_C) = \mathrm{Var}(K) + \mathrm{Var}[Q(C)] + 16\mathrm{Var}[Q(H)] + 8\mathrm{Cov}[Q(C),Q(H)] \quad (S2)$$

Here K = $D_{atom}$(CH₄)+Q(CH₄) is assumed uncorrelated with the atomic inputs. Otherwise add −2Cov[K,Q(C)]−8Cov[K,Q(H)]. A common K uncertainty changes the offset but not metal ordering; metal-specific methane adsorption uncertainty need not cancel.

Table S10. Each scenario uses 200,000 draws; NumPy default_rng seed 20260920, scenarios in table order. Gaussian 1–3 kcal mol⁻$^{1}$ denotes an independent standard deviation on each atomic input. Uniform ±10% is bounded; Gaussian 10% is a standard deviation and is unbounded. Full-order retention means $D_C$(Fe)<$D_C$(Ni)<$D_C$(Pt)<$D_C$(Cu). A sampled 100% is not a mathematical guarantee.

| Perturbation | Full-order retention | Monte Carlo standard error |
|---|---|---|
| Gaussian SD 1 kcal/mol | 100.000% | 0.000 percentage points |
| Gaussian SD 2 kcal/mol | 98.924% | 0.023 percentage points |
| Gaussian SD 3 kcal/mol | 91.062% | 0.064 percentage points |
| 10% uniform | 75.382% | 0.096 percentage points |
| 10% normal | 45.948% | 0.111 percentage points |

The unresolved model-error budget includes UBI-QEP functional form, gas/adsorbate conventions, correlated parameter errors, coverage, facet, phase and experimental comparability. The simulations quantify the declared input scenarios only; they do not estimate a confidence interval for catalyst performance.

## S7. Condition-resolved experimental evidence

Table S11. Condition-resolved experimental evidence retained by study and measurement category. Initial or maximum CH₄ conversion, retained fraction, H₂ yield, and apparent activation energy are reported separately and are not treated as interchangeable performance metrics. NR denotes information not reported in the inspected source; all percentages are source-reported unless marked as derived.

| Catalyst/record | Conditions/time basis | Numerical observation | Source |
|---|---|---|---|
| Fe/Al₂O₃ CP | 700 °C; initial | 20.8% CH₄ conversion | Fakeeha [29] |
| Fe/Al₂O₃ Imp | 700 °C; initial | 47.8% CH₄ conversion | Same study |
| Fe/Al₂O₃ Imp | 800 °C; 120 min | ≈80–85% conversion; stopped for plugging | Same study |
| FeCo 1:1:1, in situ | 700 °C; 650 min | 81.1% maximum; 97.9% of maximum retained | Cao [31] |
| FeCo 2:1:1, in situ | 700 °C; 650 min | 83.0% maximum; 92.3% of maximum retained | Cao [31] |
| FeCo 3:1:1, in situ | 700 °C; 650 min | 78.1% maximum; 98.1% of maximum retained | Cao [31] |

| | | | |
|---|---|---|---|
| FeCo 1:1:1, pre-reduced | 700 °C; 650 min | 26.1% maximum; 92.0% of maximum retained | Cao [31] |
| FeCo 2:1:1, pre-reduced | 700 °C; 650 min | 42.0% maximum; 80.2% of maximum retained | Cao [31] |
| FeCo 3:1:1, pre-reduced | 700 °C; 650 min | 36.7% maximum; 97.5% of maximum retained | Cao [31] |
| NiCu/C low-T regime | 450–600 °C study | Eapp 86 kJ mol⁻$^{1}$; CH₄ order ≈1 | Schoemaker 2023 [30] |
| NiCu/C high-T regime | Same catalyst family | Eapp 45 kJ mol⁻$^{1}$; CH₄ order ≈1.5 | Same study |
| Ni/SiO₂ microflakes | Study maximum; not normalized here | 74% maximum H₂ yield | Pudukudy [33] |

Fakeeha: 20 wt% Fe, 400 °C calcination for the matched preparation comparison; 0.3 g catalyst; atmospheric pressure; GHSV 5000 mL h⁻$^{1}$ gcat⁻$^{1}$; CH₄:N₂=1.5:1; H₂ reduction at 700 °C for 90 min. The derived preparation contrast is 27.0 percentage points, not an intrinsic rate ratio. The 800 °C run ending by plugging is not a measured deactivation lifetime.

Cao: nominal Fe:Co:Al precursor ratios shown; 70 mg starting sample; 20 sccm CH₄; ramp 5 °C min⁻$^{1}$ to 700 °C followed by the time-on-stream test. Pre-reduction used 10% H₂/Ar at 700 °C for 2 h. Pressure is NR in the inspected reaction methods. In situ and pre-reduced masses do not establish equal exposed-metal-site inventories. Maximum conversion and retention are separate observables; retained fractions must not be reported as absolute conversions. Ramp-to-800 °C maxima and thermogravimetric tests with added Ar are excluded from this isothermal comparison.

Schoemaker: the reported apparent barriers and reaction orders characterize two temperature regimes; they are not assigned to an elementary C–H cleavage. The separate 2024 NiCu/C composition study [32] supports sustained growth at moderate Cu content. The Ni/Co/Fe microflake study reports different carbon products and activity/stability behavior, but its maximum Ni hydrogen yield is not converted into CH₄ conversion. No alloy $D_C$ values are interpolated.

These twelve numerical records improve the independent evidence base, but correlated records from the same study are not twelve independent elemental descriptor values. A universal rate/lifetime regression would require comparable site normalization, catalyst state, feed, temperature and uncertainty. Missing metadata and absence of such a harmonized database is explicit limitations.
