# Supplementary material for: Evaluating carbon affinity in catalytic methane decomposition using UBI-QEP energetics and DFT carbon-binding analysis
Source: RSC Adv. 2026 Oct 20. Online ahead of print. doi: 10.1039/d6ra05232k (PMC13681386; doi:10.1039/d6ra05232k)
Supplement: Supplementary file 1 [file RA-OLF-D6RA05232K-s001.pdf]

Supplementary Information (SI) for RSC Advances.
This journal is © The Royal Society of Chemistry

Supporting information

Evaluating Carbon Affinity in Catalytic Methane Decomposition Using UBI-QEP Energetics and DFT Carbon-Binding Analysis

Yahya Sorkhe[1,2]

[1]Department of Metallurgical and Chemical Engineering, Istanbul Technical University, Istanbul 34469, Turkey.

[2] Faculty of science, Koc University, Istanbul, Turkey.

Corresponding author: Sorkhe20@itu.edu.tr

This Supporting Information provides the provenance and recalculation of the UBI-QEP dataset, independently recalculated statistics, verified external DFT inputs, thermodynamic calculations, and condition-resolved experimental evidence.

S1. Provenance and recalculation of the UBI-QEP dataset.

The UBI-QEP analysis used in this work distinguishes between published chemisorption input parameters and quantities recalculated in the present study. The chemisorption energies Q(C), Q(H), Q(CH), Q(CH_2), and Q(CH_3) for Fe, Ni, Pt, and Cu were taken from the published UBI-QEP treatment of methane-related surface chemistry. Using these published chemisorption inputs, the gas-phase C–H dissociation energies were independently recalculated from standard gas-phase enthalpies of formation obtained from the NIST Chemistry WebBook. These gas-phase quantities were then used together with the UBI-QEP expression to recalculate the intrinsic reaction barriers for the successive CH_x^* □CH_{x-1}^* + H^* steps. The recalculated R3–R5 barriers closely reproduce the corresponding published values. Small differences arise from rounding of the historical chemisorption parameters and the use of independently tabulated gas-phase thermochemical data. The R2 step additionally requires the methane adsorption energy Q(CH_4), which is not independently specified in the retained input table. Table S1 lists the published chemisorption inputs and their provenance. Table S2 provides the gas-phase thermochemical quantities used in the recalculation. Table S3 compares the recalculated UBI-QEP barriers with the published values.

Table S1. Provenance of the numerical quantities used in the UBI-QEP analysis.

Quantity	Metals / system	Origin	Use in this work
Q(C), Q(H), Q(CH), Q(CH_2), Q(CH_3)	Fe, Ni, Pt, Cu	Hei et al. [17]	Published input parameters

UBI-QEP analytic formulation	General	Shustorovich and Sellers [16]	Barrier and reaction-energy equations
Gas-phase ΔfH° values	CH_4, CH_3, CH_2, CH, C(g), H(g)	NIST Chemistry WebBook	Calculation of C-H dissociation energies
R3-R5 intrinsic barriers	Fe, Ni, Pt, Cu	Recalculated in this work	Compared with published UBI-QEP values
R2 intrinsic barriers	Fe, Ni, Pt, Cu	Hei et al. [17]	Retained because Q(CH_4) is not independently available in the retained input set
DC and descriptor correlations	Fe, Ni, Pt, Cu	Recalculated / reanalysed in this work	Descriptor analysis
DFT C adsorption	14 metals	Piqué et al. [22]	External energetic comparison
Migration barriers	Fe, Ni, Pt, Cu	Piqué et al. [22]	Transport comparison

Table S2. Published UBI-QEP chemisorption inputs used in the present recalculation. Positive Q values denotes exothermic chemisorption. Values are used as fixed literature inputs; no refitting or modification was performed. (Units: kcal mol−1).

Metal	Q(C)	Q(CH)	Q(CH_2)	Q(CH_3)	Q(H)
Fe	200.0	142.3	104.4	58.1	63.5
Ni	171.0	116.0	82.6	44.9	63.0
Pt	150.0	97.4	67.6	36.1	61.0
Cu	120.0	71.6	47.5	24.6	56.0

S1.1 Gas-phase C–H dissociation energies

The gas-phase bond dissociation energy for each successive methane dehydrogenation step was calculated from standard gas-phase enthalpies of formation according to

$$D_{gas}(CH_x\text{-}H)=\square_f H^\circ(CH_{x-1})+\square_f H^\circ(H)-\square_f H^\circ(CH_x)$$

Standard gas-phase enthalpies of formation were taken from the NIST Chemistry WebBook at approximately 298 K.

Table S3. Gas-phase C-H dissociation energies used in the UBI-QEP recalculation.

Step	Gas-phase reaction	Dgas / kcal mol−1
R2	CH_4 → CH_3 + H	104.82
R3	CH_3 → CH_2 + H	109.63
R4	CH_2 → CH + H	101.75
R5	CH → C + H	81.39

S1.2 Recalculation of intrinsic UBI-QEP barriers

For the formal surface dissociation

$$CH_x^* → CH_{x\text{-}a}^* + H^*$$

The intrinsic barrier was recalculated using:

$$E_a = \frac{1}{2}[D_{gas} + Q_{reactant} - Q_{product} - Q(H) + \frac{Q_{product}Q(H)}{Q_{product} + Q(H)}$$

Here, $Q_{reactant}$ and $Q_{product}$ are the published UBI-QEP chemisorption energies listed in Table S2, and D_{gas} is the corresponding gas-phase C–H dissociation energy from Table S3. For example, for the Fe R3 surface dissociation step, *CH□* □ CH₂* + H**, the published UBI-QEP chemisorption energies are Q(CH₃) = 58.1 kcal mol⁻^{1}, Q(CH₂) = 104.4 kcal mol⁻^{1}, and Q(H) = 63.5 kcal mol⁻^{1}. The corresponding gas-phase C–H dissociation energy is 109.63 kcal mol⁻^{1}. Substitution of these values into the UBI-QEP barrier expression gives: Ea = 19.66 kcal mol⁻^{1} = 82.25 kJ mol⁻^{1}. The published value for this step is 83 kJ mol⁻^{1}, showing close agreement with the recalculated result. The small difference is consistent with rounding of the historical input parameters and the use of independently tabulated gas-phase thermochemical data.

Table S4. Comparison of recalculated and published UBI-QEP intrinsic barriers.

Metal	Step	Recalculated / kJ mol−1	Published / kJ mol−1	Difference / kJ mol−1
Fe	R3	82.25	83	−0.75
Fe	R4	92.59	92	+0.59
Fe	R5	17.55	17	+0.55
Ni	R3	93.45	95	−1.55
Ni	R4	96.61	96	+0.61
Ni	R5	19.73	19	+0.73
Pt	R3	102.92	104	−1.08

Pt	R4	101.38	100	+1.38
Pt	R5	23.34	23	+0.34
Cu	R3	118.06	119	−0.94
Cu	R4	111.04	110	+1.04
Cu	R5	31.74	31	+0.74

The small deviations, all within approximately 1.6 kJ mol^{-1}, are consistent with rounding of the historical chemisorption parameters and differences in the tabulated gas-phase thermochemical values. These comparisons confirm that the published UBI-QEP barrier sequence can be reproduced from the stated input data and analytic expression.

S2. Descriptor identity and statistics

Σ ΔHₓ = Q(CH₄) - Q(C) + D_{atom}(CH₄) - 4Q(H) (S1)

All Q(CH₃), Q(CH₂) and Q(CH) terms cancel in the sum. The result follows from Hess's law without quasi-equilibrium. The reported D_C values and the combined atomic inputs give the following closure check.

Table S5. The common-term mean is 1684.290 kJ mol⁻^{1} and its range is 0.704 kJ mol⁻^{1}. This term is back-calculated, not independently measured. Relative input-based differences from Fe are 129.704, 251.040 and 460.240 kJ mol⁻^{1}; reported rounded differences are 129, 251 and 460 kJ mol⁻^{1}.

Metal	Q(C)+4Q(H) / kcal mol⁻^{1}	Inferred common term / kJ mol⁻^{1}
Fe	454.0	1684.536
Ni	423.0	1683.832
Pt	394.0	1684.496
Cu	344.0	1684.296

Table S6. Pearson coefficients use unweighted pairs; Spearman coefficients use their ranks. R^2 is r^2 for ordinary least squares with an intercept, not agreement with the identity line. No significance or out-of-sample prediction claim is made. Descriptor correlations are algebraically dependent; the DFT inputs are externally sourced.

Comparison	n	Pearson r	OLS R^2	Spearman ρ
**D_C versus Q(C)**	4	-0.993979047	0.987994346	-1.000
**D_C versus Q(C)+4Q(H)**	4	-0.999998639	0.999997279	-1.000
Q(C) versus signed DFT Eads	4	-0.928517985	0.862145649	-0.800

S3. Endpoint and gas-phase thermochemical reference

Twice adding 2H* → H₂(g) + 2*, with enthalpy 2Q(H) − D(H₂), and referencing methane to the gas phase gives D_{atom}(CH₄) − 2D(H₂) − Q(C). Hydrogen adsorption therefore cancels exactly from that endpoint, while hydrogen still influences kinetics and gas chemical potential.

NIST formation enthalpies near 298 K [26] give 716.67, 218.00 and −74.873 kJ mol⁻^{1} for C(g), H(g) and CH₄(g), respectively. Thus D_{atom}(CH₄) = 1663.543 kJ mol⁻^{1} and D(H₂) = 436.00 kJ mol⁻^{1}. Combining these with the mean original common term would imply Q(CH₄) ≈ 20.75 kJ mol⁻^{1}. This inferred value is not substituted for an independently sourced methane adsorption parameter.

An absolute C* free-energy boundary would require consistent electronic energies, zero-point and vibrational terms, adsorbate coverage and reference-state conversion. Graphite equilibrium is calculated separately; gas-atom entropy is not assigned directly to an adsorbed atom.

S4. Verified 14-metal DFT benchmark

Source: Piqué et al. [22], supporting file cs2c01562_si_001.pdf, retrieved through the public Europe PMC supplementary-files service for PMC9880994. Tables S1–S3 were checked as PDF tables and extracted text. Their positive adsorption binding convention is converted here to Eads = E(slab+C) − E(slab) − E(Catom). Select the largest surface Eads in the source for the stated facet; never select a subsurface Eabs instead.

Table S7. Surface binding magnitudes B and signed adsorption energies. Conversion: 1 eV per particle = 96.48533212 kJ mol⁻^{1}.

Metal	Facet	Selected surface site	B / kJ mol⁻^{1}	Eads / eV
V	bcc(110)	hollow	806	-8.353601

Cr	bcc(110)	hollow	750	-7.773202
Fe	bcc(110)	hollow	894	-9.265657
Co	hcp(0001)	hollow	660	-6.840418
Ni	fcc(111)	hcp hollow	651	-6.747140
Cu	fcc(111)	fcc hollow	440	-4.560279
Nb	bcc(110)	hollow	770	-7.980488
Mo	bcc(110)	hollow	756	-7.835388
Ru	hcp(0001)	hollow	718	-7.441546
Rh	fcc(111)	hcp hollow	706	-7.317174
Pd	fcc(111)	hcp hollow	682	-7.068432
W	bcc(110)	hollow	763	-7.907938
Ir	fcc(111)	hcp hollow	720	-7.462274
Pt	fcc(111)	fcc hollow	707	-7.327539

Protocol: VASP, PAW, PBE; 415 eV plane-wave cutoff; six layers, lower three fixed; 3 × 3 × 1 k-point mesh; dilute C coverages 1/9 or 1/8 monolayer; isolated atom reference. Fe, Co and Ni are spin-polarized; Cr is not. Vacuum gaps and slab cell geometry differ as specified in the primary methods. These choices constitute one consistent computational study but do not remove facet, coverage or magnetic-model limitations. This PBE table is not mixed numerically with the revised-PBE calculations of Pototschnig et al.

Table S8. Core comparison and facet variation. Facet ranges are structural ranges, not calibrated uncertainties.

**Metal**	**Q(C) / kJ mol⁻^{1}**	**Dense-facet B / kJ mol⁻^{1}**	**B range over three facets**
Fe	836.800	894	748–894
Ni	715.464	651	651–792
Pt	627.600	707	662–751
Cu	502.080	440	440–584

Binding-magnitude r = 0.928517985, R^2 = 0.862145648 and ρ = 0.800. Removing Cu gives r = 0.794057735. Five of six pairwise orders agree; Ni/Pt is the inversion. The fourteen-metal context does not increase the number of paired empirical inputs beyond four. No elemental UBI-QEP parameters are inferred from DFT.

Manuscript Table 4 reproduces the four dense-facet rows from source Table S10. Path labels are Fe: b6/b7/b8/b9; Ni: a13/a14/a17/a19; Pt and Cu: a12/a15 (Pt) or a14 (Cu)/a17/a19 for surface, subsurface, entry and emergence, respectively. Different local-minimum paths should not be collapsed into one equilibrium two-state model. No filament probabilities are derived.

S5. Temperature, pressure and equilibrium

Table S9. The NIST-JANAF standard pressure is 0.1 MPa = 1 bar. Methane formation is (graphite)+2H₂(g)→CH₄(g); decomposition ΔG° has the opposite sign [27].

T / K	ΔG° / kJ mol⁻¹	Kp	Xeq, 1 bar
700	+12.643	0.113916	0.166405
800	+2.115	0.727625	0.392313
900	-8.616	3.162641	0.664490
1000	-19.492	10.426480	0.850137
1100	-30.472	27.987638	0.935389
1200	-41.524	64.188521	0.970226
1300	-52.626	130.165923	0.984980

For one mole initial CH₄, gas amounts are 1−X methane and 2X hydrogen, giving yCH₄=(1−X)/(1+X) and yH₂=2X/(1+X). At fixed total pressure, $Kp=4(P/p^{\circ})X^2/(1-X^2)$. This calculation excludes initial hydrogen and inert gas. The zero of linearly interpolated ΔG° is approximately 819.7 K, and Xeq at Kp=1 and P=p° is 0.447214. Neither result is a kinetic onset criterion.

S6. Uncertainty and sensitivity

$$\mathrm{Var}(D_C) = \mathrm{Var}(K) + \mathrm{Var}[Q(C)] + 16\mathrm{Var}[Q(H)] + 8\mathrm{Cov}[Q(C),Q(H)] \quad \text{(S2)}$$

Here K = D_{atom}(CH₄)+Q(CH₄) is assumed uncorrelated with the atomic inputs. Otherwise add −2Cov[K,Q(C)]−8Cov[K,Q(H)]. A common K uncertainty changes the offset but not metal ordering; metal-specific methane adsorption uncertainty need not cancel.

Table S10. Each scenario uses 200,000 draws; NumPy default_rng seed 20260920, scenarios in table order. Gaussian 1–3 kcal mol^{-1} denotes an independent standard deviation on each atomic input. Uniform ±10% is bounded; Gaussian 10% is a standard deviation and is unbounded. Full-order retention means $D_C(Fe)<D_C(Ni)<D_C(Pt)<D_C(Cu)$. A sampled 100% is not a mathematical guarantee.

Perturbation	Full-order retention	Monte Carlo standard error
Gaussian SD 1 kcal/mol	100.000%	0.000 percentage points
Gaussian SD 2 kcal/mol	98.924%	0.023 percentage points
Gaussian SD 3 kcal/mol	91.062%	0.064 percentage points
10% uniform	75.382%	0.096 percentage points
10% normal	45.948%	0.111 percentage points

The unresolved model-error budget includes UBI-QEP functional form, gas/adsorbate conventions, correlated parameter errors, coverage, facet, phase and experimental comparability. The simulations quantify the declared input scenarios only; they do not estimate a confidence interval for catalyst performance.

S7. Condition-resolved experimental evidence

Table S11. Condition-resolved experimental evidence retained by study and measurement category. Initial or maximum CH_4 conversion, retained fraction, H_2 yield, and apparent activation energy are reported separately and are not treated as interchangeable performance metrics. NR denotes information not reported in the inspected source; all percentages are source-reported unless marked as derived.

Catalyst/record	Conditions/time basis	Numerical observation	Source
Fe/Al_2O_3 CP	700 °C; initial	20.8% CH_4 conversion	Fakeeha [29]
Fe/Al_2O_3 Imp	700 °C; initial	47.8% CH_4 conversion	Same study
Fe/Al_2O_3 Imp	800 °C; 120 min	≈80–85% conversion; stopped for plugging	Same study
FeCo 1:1:1, in situ	700 °C; 650 min	81.1% maximum; 97.9% of maximum retained	Cao [31]
FeCo 2:1:1, in situ	700 °C; 650 min	83.0% maximum; 92.3% of maximum retained	Cao [31]
FeCo 3:1:1, in situ	700 °C; 650 min	78.1% maximum; 98.1% of maximum retained	Cao [31]

FeCo 1:1:1, pre-reduced	700 °C; 650 min	26.1% maximum; 92.0% of maximum retained	Cao [31]
FeCo 2:1:1, pre-reduced	700 °C; 650 min	42.0% maximum; 80.2% of maximum retained	Cao [31]
FeCo 3:1:1, pre-reduced	700 °C; 650 min	36.7% maximum; 97.5% of maximum retained	Cao [31]
NiCu/C low-T regime	450–600 °C study	Eapp 86 kJ mol⁻^{1}; CH₄ order ≈1	Schoemaker 2023 [30]
NiCu/C high-T regime	Same catalyst family	Eapp 45 kJ mol⁻^{1}; CH₄ order ≈1.5	Same study
Ni/SiO₂ microflakes	Study maximum; not normalized here	74% maximum H₂ yield	Pudukudy [33]

Fakeeha: 20 wt% Fe, 400 °C calcination for the matched preparation comparison; 0.3 g catalyst; atmospheric pressure; GHSV 5000 mL h⁻^{1} gcat⁻^{1}; CH₄:N₂=1.5:1; H₂ reduction at 700 °C for 90 min. The derived preparation contrast is 27.0 percentage points, not an intrinsic rate ratio. The 800 °C run ending by plugging is not a measured deactivation lifetime.

Cao: nominal Fe:Co:Al precursor ratios shown; 70 mg starting sample; 20 sccm CH₄; ramp 5 °C min⁻^{1} to 700 °C followed by the time-on-stream test. Pre-reduction used 10% H₂/Ar at 700 °C for 2 h. Pressure is NR in the inspected reaction methods. In situ and pre-reduced masses do not establish equal exposed-metal-site inventories. Maximum conversion and retention are separate observables; retained fractions must not be reported as absolute conversions. Ramp-to-800 °C maxima and thermogravimetric tests with added Ar are excluded from this isothermal comparison.

Schoemaker: the reported apparent barriers and reaction orders characterize two temperature regimes; they are not assigned to an elementary C–H cleavage. The separate 2024 NiCu/C composition study [32] supports sustained growth at moderate Cu content. The Ni/Co/Fe microflake study reports different carbon products and activity/stability behavior, but its maximum Ni hydrogen yield is not converted into CH₄ conversion. No alloy D_C values are interpolated.

These twelve numerical records improve the independent evidence base, but correlated records from the same study are not twelve independent elemental descriptor values. A universal rate/lifetime regression would require comparable site normalization, catalyst state, feed, temperature and uncertainty. Missing metadata and absence of such a harmonized database is explicit limitations.
